# Rewriting cellular fate: epigenetic interventions in obesity and cellular programming

**DOI:** 10.1186/s10020-024-00944-2

**Published:** 2024-10-10

**Authors:** Rui-lin Li, Sheng Kang

**Affiliations:** Department of Cardiology, Shanghai East Hospital, School of Medicine, Tongji University, Jimo Road 150, Shanghai, 200120 China

**Keywords:** Epigenetics, Cellular programming, Reprogramming

## Abstract

External constraints, such as development, disease, and environment, can induce changes in epigenomic patterns that may profoundly impact the health trajectory of fetuses and neonates into adulthood, influencing conditions like obesity. Epigenetic modifications encompass processes including DNA methylation, covalent histone modifications, and RNA-mediated regulation. Beyond forward cellular differentiation (cell programming), terminally differentiated cells are reverted to a pluripotent or even totipotent state, that is, cellular reprogramming. Epigenetic modulators facilitate or erase histone and DNA modifications both in vivo and in vitro during programming and reprogramming. Noticeably, obesity is a complex metabolic disorder driven by both genetic and environmental factors. Increasing evidence suggests that epigenetic modifications play a critical role in the regulation of gene expression involved in adipogenesis, energy homeostasis, and metabolic pathways. Hence, we discuss the mechanisms by which epigenetic interventions influence obesity, focusing on DNA methylation, histone modifications, and non-coding RNAs. We also analyze the methodologies that have been pivotal in uncovering these epigenetic regulations, i.e., Large-scale screening has been instrumental in identifying genes and pathways susceptible to epigenetic control, particularly in the context of adipogenesis and metabolic homeostasis; Single-cell RNA sequencing (scRNA-seq) provides a high-resolution view of gene expression patterns at the individual cell level, revealing the heterogeneity and dynamics of epigenetic regulation during cellular differentiation and reprogramming; Chromatin immunoprecipitation (ChIP) assays, focused on candidate genes, have been crucial for characterizing histone modifications and transcription factor binding at specific genomic loci, thereby elucidating the epigenetic mechanisms that govern cellular programming; Somatic cell nuclear transfer (SCNT) and cell fusion techniques have been employed to study the epigenetic reprogramming accompanying cloning and the generation of hybrid cells with pluripotent characteristics, etc. These approaches have been instrumental in identifying specific epigenetic marks and pathways implicated in obesity, providing a foundation for developing targeted therapeutic interventions. Understanding the dynamic interplay between epigenetic regulation and cellular programming is crucial for advancing mechanism and clinical management of obesity.

## Introduction

Epigenetics refers to the heritable changes in gene expression or cellular phenotype without alterations in the DNA sequence, involving chemical modifications to DNA and various RNAs. The epigenome’s patterns are influenced by external constraints such as development, disease, and the environment, interacting with the underlying DNA sequence (Huang et al. 2024). Epigenetics focuses on the regulation of when and where specific genes are expressed, while epigenomics involves analyzing genetic modifications on a cellular or organismal scale, and epigenetic modifications play a crucial role in cellular differentiation, gene regulation, and the development of various pathological conditions. Given the rapid accumulation of genome-wide epigenomic modification maps across cellular differentiation process, it is necessary to characterize epigenetic dynamics and reveal their impacts on differential gene regulation, i.e., DiffEM, a computational method for differential analysis of epigenetic modifications, could identify highly dynamic modification sites along cellular differentiation process (Zhang et al. 2019).

A deeper understanding of the Waddington energy landscape of embryogenesis and cell reprogramming processes at single-cell resolution as characterized by recent studies, provides that cell fate decision is progressively specified in a continuous process. Moreover, the transition of cells from one steady state to another in embryogenesis and cell reprogramming processes was dynamically simulated on the energy ladder (Li et al. 2023). Deciphering the decisive factors that drive fate bifurcations in somatic cell reprogramming is essential for harnessing the therapeutic potential of regenerative medicine. The identification of specific signaling pathways and epigenetic modifiers that dictate the transition between cell states can inform strategies for redirecting cellular identity (Long et al. 2023).

Noticeably, obesity is a growing public health challenge worldwide, the World Health Organization reports that obesity has tripled in the last 50 years. In 2016, more than 650 million adults aged 18 years and older were obese. It is estimated that by 2030, approximately 20% of the world population will be obese, and 38% will be overweight (Yadav and Jawahar 2023). The rapid increase in obesity rates is evident not only in high-income countries but also in low- and middle-income countries, making it a global epidemic with serious health consequences.

Recent advances in determining the regulatory mechanisms reveal that the compromised epigenomes are molecularly interlinked to altered *cis*-regulatory element activity and chromosome architecture in the adipose tissue. Further, the emerging roles of DNA methylation in the maintenance of 3D chromosome conformation and its pathophysiological significance concern adipose tissue function (Park et al. 2021). The different environmental cues can epigenetically reprogram adipocyte fate and function, mainly by altering DNA methylation and histone modification patterns. Intriguingly, it appears that transcription factors and chromatin-modifying coregulator complexes are the key regulatory components that coordinate both signaling-induced transcriptional and epigenetic alterations in adipocytes (Barilla et al. 2021). Developmental pluripotency-associated 2 (Dppa2) and developmental pluripotency-associated 4 (Dppa4) as positive drivers were helpful for transcriptional regulation of zygotic genome activation (ZGA). Moreover, the discovery that Dppa2/4 can act as a trigger for signaling pathways, promoting zygote genome activation by binding to CG-rich regions, highlights the intricate interplay between epigenetic regulators and genomic elements during early developmental stages (Li et al. 2021). This finding is particularly relevant to understanding the initiation of cellular programming and the establishment of epigenetic marks that may predispose individuals to obesity.

The pathogenesis of obesity involves complex interactions between genetic predisposition, environmental factors, and metabolic processes. Key metabolic pathways include those related to lipid metabolism, insulin signaling, and energy homeostasis. Genes such as FTO (fat mass and obesity-associated gene), LEP (leptin), MC4R (melanocortin 4 receptor), and PPARG (peroxisome proliferator-activated receptor gamma) are well-established contributors to obesity susceptibility. These genes influence processes like appetite regulation, adipogenesis, and energy expenditure, leading to disruptions in energy balance and increased fat storage (Serra-Juhé et al. 2020; Sarzynski et al. 2011).

## Epigenetic modifications and their impact

Epigenetic modifications influence gene transcription and post-transcriptional regulation through various mechanisms. Studies have shown that parental environmental factors affect offspring gene expression through DNA methylation, histone covalent modifications, and chromatin remodeling, for example, the huge health burden accompanying obesity is not only attributable to inadequate dietary and sedentary lifestyle habits, since it is found that a predisposing genetic make-up and other putative determinants concerning easier weight gain and fat deposition (Martínez et al. 2012). In addition, in conjunction with histone modifications, DNA methylation plays critical roles in gene silencing through chromatin remodeling, which is also interconnected with the DNA damage response, maintenance of stem cell properties, and cell differentiation programs (Bariar et al. 2013). These modifications have long-lasting effects on gene expression patterns and cellular function, potentially influencing an individual's susceptibility to various diseases later in life.

DNA methylation, one of the most well-studied epigenetic modifications, involves the addition of a methyl group to cytosine residues in CpG dinucleotides (Valente et al. 2023). This modification is generally associated with gene silencing and plays a crucial role in genomic imprinting, X-chromosome inactivation, and the regulation of tissue-specific gene expression (Waggoner 2007). The enzymes responsible for DNA methylation, known as DNA methyltransferases (DNMTs), are essential for maintaining methylation patterns during cell division and establishing new methylation marks during development, i.e., the unique regions of the methylated genome by specific DNMT isoforms and its potential for dietary intervention to modify the epigenome (Sae-Lee et al. 2022).

Histone modifications represent another important class of epigenetic regulators. These covalent modifications to histone proteins can alter chromatin structure and accessibility, thereby influencing gene expression. Common histone modifications include acetylation, methylation, phosphorylation, and ubiquitination. The combination of these modifications, often referred to as the "histone code," can either promote or repress gene transcription depending on the specific marks and their location (Jeffers 2024).

RNA-mediated regulation involves various types of non-coding RNAs that play crucial roles in gene expression and cellular function. These include small RNAs (sRNAs), non-coding RNAs (ncRNAs), microRNAs (miRNAs), short interfering RNAs (siRNAs), PIWI-interacting RNAs (piRNAs), antisense RNAs, riboswitches, RNA methylation, editing, and splicing (Jiang et al. 2017; Haggarty 2015). MicroRNAs, in particular, have emerged as important regulators of gene expression, acting post-transcriptionally to fine-tune protein levels in various cellular processes (Xu et al. 2024).

Genetic polymorphisms in DNA processing enzymes, such as DNA methyltransferases and ten-eleven translocation (TET) methylcytosine dioxygenases, also impact epigenetic states (Sharma and Rando 2017). These variations can lead to differences in epigenetic patterns between individuals and may contribute to disease susceptibility or resistance.

## Epigenetic plasticity and environmental influences

Epigenetic modifications typically occurred during terminal differentiation into somatic cells; however, these cells possessed the ability to reprogram their epigenomes in response to environmental challenges like maternal stress (McCaughan et al. 2012). Such changes induced organisms more adaptive or less suited to future challenges. This plasticity of the epigenome allows for rapid adaptation to environmental changes but can also lead to maladaptive responses in certain conditions. Epigenetic variations contributed to the onset of diseases, including cancer, neurological disorders, cardiovascular diseases, metabolic syndromes, immune disorders, and aging.

**Fig. 1:**
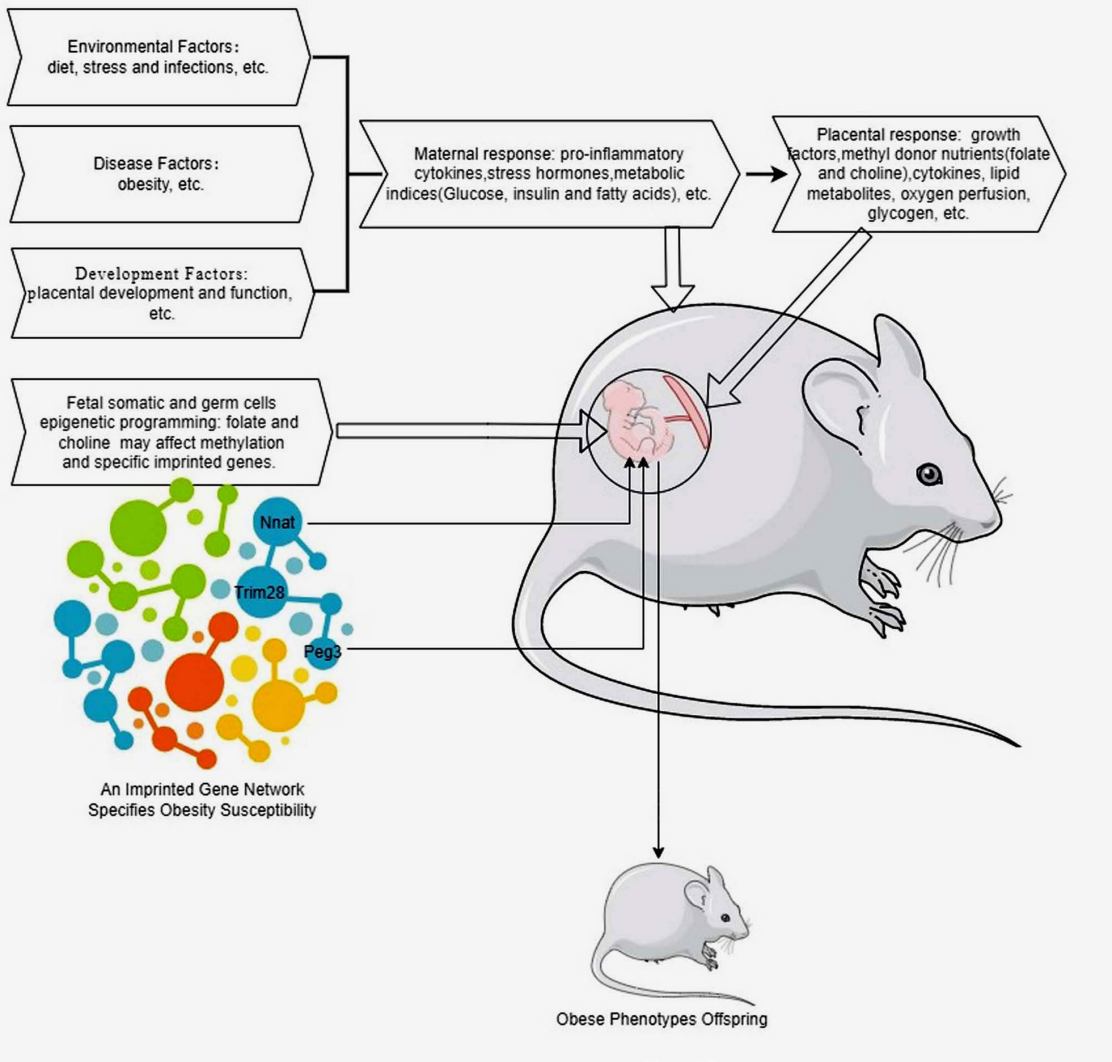
The impact of environmental factors on epigenetic programming for obesity. Environmental factors like poor maternal diet, chronic stress, or infection can disrupt the endocrine system, increasing pro-inflammatory cytokines, stress hormones, and metabolic changes (glucose, insulin, free fatty acids). These maternal responses also impact placental development and function, changing energy metabolism, lipid metabolism, oxidative stress, growth factors, and methyl donors (folate, choline). Additionally, Trim28 haploinsufficiency causes obesity by disrupting Peg3 and Nnat in an imprinted gene network. These factors, individually or combined, influence fetal somatic and germ cell epigenetic programming

Notably, through epigenetic modifications, there may be infinite developmental benefits or harms for the fetus and newborn later on in adult life health status, e.g., obesity (Fig. 1). The concept of developmental origins of health and disease (DOHaD) emphasizes the importance of early life experiences and environmental exposures in shaping long-term health outcomes (Frankenhuis et al. 2018). Regarding obesity, epigenetic modifications have been shown to play a significant role in the regulation of energy metabolism, appetite control, and adipocyte differentiation, for example, studies have demonstrated that maternal nutrition during pregnancy can influence the epigenetic programming of offspring metabolism, potentially predisposing them to obesity and related metabolic disorders later in life (Sinha et al. 2021). Specific epigenetic marks, such as DNA methylation patterns in genes involved in energy homeostasis, have been associated with obesity risk and metabolic dysfunction (Zhang et al. 2021).

## Cellular programming and obesity

It is concerned the cellular programming and obesity under the unifying umbrella of epigenetic regulation. Firstly, adipogenesis, the process by which preadipocytes differentiate into adipocytes, is a tightly regulated event influenced by epigenetic modifications. Aberrant epigenetic regulation can lead to an imbalance in adipocyte differentiation and function, contributing to obesity. Dysregulation in cellular programming can lead to an excess of adipocyte formation, contributing to obesity, such as leptin can regulate Plin5 M6A methylation by promoting FTO to affect the lipid metabolism and energy consumption (Wei et al. 2021). This includes hyperplasia (increase in fat cell number) and hypertrophy (increase in fat cell size).

Secondly, there is growing evidence to suggest that epigenetic marks can be inherited across generations, potentially influencing the susceptibility to obesity, i.e., Maternal obesity enhanced Zfp423 expression and adipogenic differentiation during fetal development, at least partially through reducing DNA methylation in the Zfp423 promoter, which is expected to durably elevate adipogenic differentiation of progenitor cells in adult tissue, programming adiposity and metabolic dysfunction later in life (Yang et al. 2013).

Thirdly, cellular reprogramming technologies, such as the induction of pluripotency, offer a promising avenue for reversing the pathogenic programming associated with obesity. By reprogramming white adipose tissue (WAT) to a brown adipose tissue (BAT)-like state, it is possible to enhance energy expenditure and reduce adiposity (Boström et al. 2012). This approach improves our understanding of epigenetic regulation to modulate metabolic health.

Fourthly, the development of targeted epigenetic drugs, such as DNA methyltransferase inhibitors and histone deacetylase inhibitors, provides a novel therapeutic strategy for obesity management. These compounds can modulate the epigenetic landscape of adipose tissue, potentially normalizing aberrant gene expression patterns that contribute to obesity, i.e., miRNA-seq analysis of brown fat revealed a strong role for miRNAs in the downregulation of central metabolic processes necessary for metabolic rate suppression, and highlighted miRNAs that could be inhibited by antagomiRs to promote brown fat activity in potential obesity treatments, or that could be used to replicate torpor in non-hibernating mammals (Logan and Storey 2021).

Fifthly, the identification of epigenetic biomarkers in obesity offers a window into the molecular mechanisms underlying this condition. For instance, differential DNA methylation at specific loci has been associated with obesity and related metabolic disorders, providing a potential diagnostic and therapeutic target, and Andrade et al. reported that DNA methylation patterns can potentially discriminate between metabolically unhealthy overweight/obesity (MUHO) and metabolically healthy overweight/obesity (MHO), then provide new clues into why some people with obesity are less susceptible to dysglycemia (Andrade et al. 2021).

## Reprogramming and the potential for reversing obesity

**Fig. 2:**
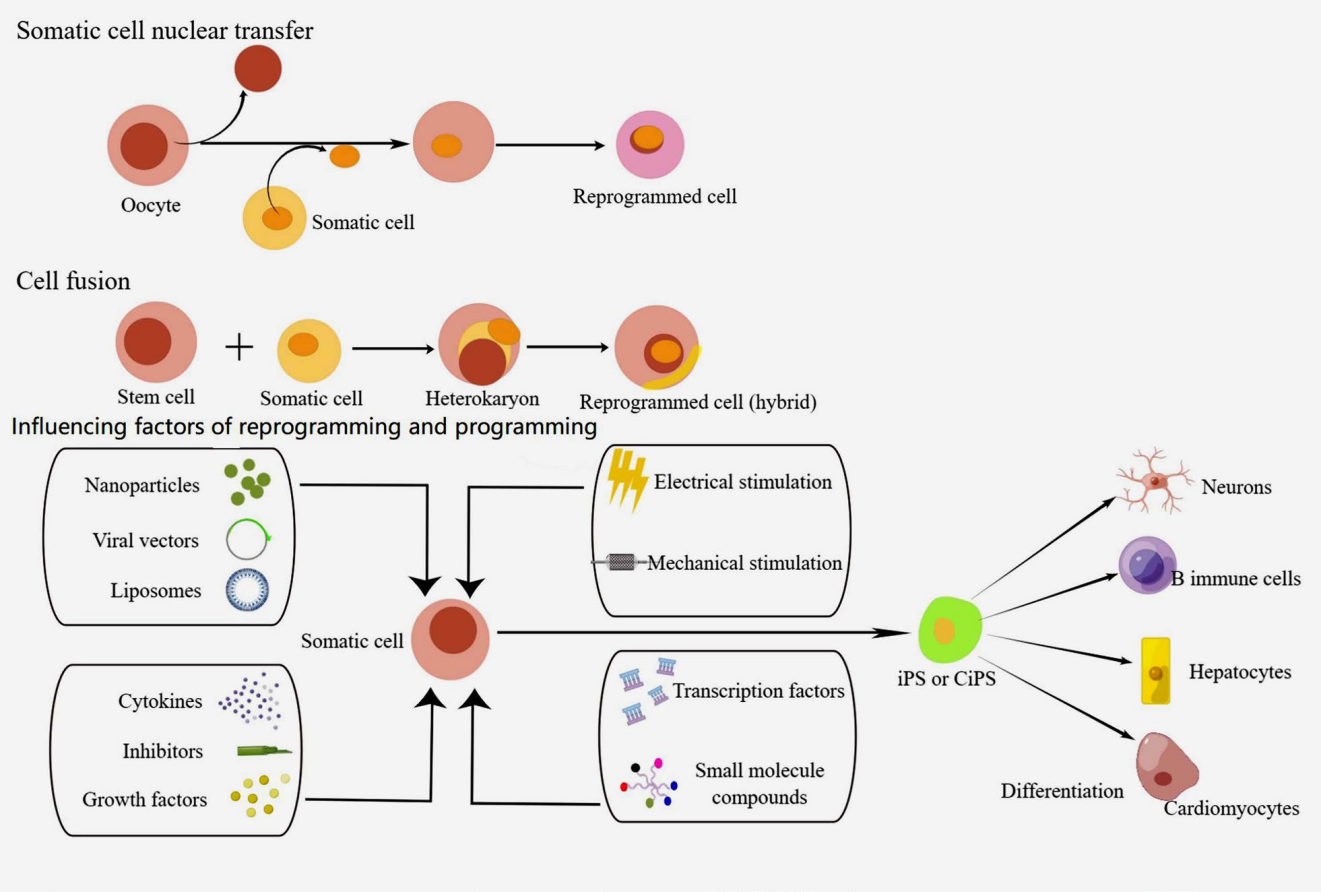
Reprogramming somatic cells to a pluripotent state and differentiation into functional cell types. In somatic nuclear transfer, a somatic cell nucleus is reprogrammed by placing it into an enucleated egg, using factors in the egg’s cytoplasm, which can lead to cloning. In cell fusion, somatic cells fuse with stem cells, creating hybrids with a pluripotent phenotype, shown by reactivation of pluripotency genes and the ability to form chimeric embryos. In inducing pluripotency, somatic cells are reprogrammed into iPSCs or CiPSCs, which can differentiate into functional cell types (neurons, B cells, hepatocytes, cardiomyocytes). Methods include microRNA delivery (nanoparticles, viral vectors, liposomes), paracrine signals (cytokines, inhibitors, growth factors), physical stimuli (electrical, mechanical), small-molecule compounds, and gene transcription factors (Oct4, Sox2, Klf4, C-myc)

Cellular reprogramming refers to the process of changing a cell’s identity, often involving the erasure or modification of established epigenetic marks. Techniques used in cellular reprogramming include the induction of pluripotency, where differentiated cells are reverted to a more stem cell-like state (Fig. 2). In the context of obesity, reprogramming strategies focus on reversing the pathogenic adipogenic programming. For example, inducing a shift from WAT, which stores fat, to beige or BAT, which burns fat through thermogenesis, is a promising therapeutic avenue (Ong et al. 2020). This involves reprogramming cells to express genes associated with BAT-like functions, for instance, glutamine activates thermogenic adipocyte differentiation and uncovers an unexpected role of C/EBPb-Prdm9-mediated H3K4me3 and transcriptional reprogramming in adipocyte differentiation and thermogenesis (Pan et al. 2023).

Epigenetic reprogramming techniques also aim to alter the expression of obesity-associated genes, potentially reducing adiposity and improving metabolic profiles (Dalgaard et al. 2016). Interventions may target specific histone modifications, non-coding RNAs and key genes involved in adipogenesis (Table 1).

**Table 1:** Epigenetic interventions and approaches in obesity management

Epigenetic target	Mechanism/Pathway	Therapeutic strategy	Potential outcomes	References
DNA Methylation	Aberrant methylation patterns in promoters of obesity-related genes (e.g., POMC, FTO)	DNA methyltransferase inhibitors (e.g., 5-azacytidine); Nutritional modulation (e.g., folate, vitamin B12)	Restoring normal gene expression and improving metabolic function	Yao et al. (2020), Melnik et al. (2024); Kuroda et al. (2016); Daviddi et al. (2020)
Histone Modifications	Dysregulation of histone acetylation (e.g., HDAC9) in dipogenesis-related genes (e.g., ABCG1)	HDAC9 deleted; Activators of HATs for balanced chromatin states	Altered adipocyte differentiation and reduced fat storage	Chatterjee et al. (2011), Khamis et al. (2020); Ong et al. (2020)
Non-coding RNAs	miRNA-mediated regulation of genes in lipid metabolism and inflammation (e.g., miR-33, miR-27)	miRNA mimics or inhibitors (antagomiRs); Targeted delivery systems (e.g., lipid nanoparticles)	Modulating adipocyte function and reducing systemic inflammation	Can et al. (2016), Logan et al. (2021), El-Araby et al. (2024)
Chromatin Remodeling Complexes	Misregulation of SWI/SNF complexes in energy balance and liver steatosis (e.g., BAF60b)	Targeting specific ATPase subunits (e.g., circulating NEFA); Small molecule inhibitors for chromatin remodeling	Enhanced metabolic efficiency and insulin responsiveness	Zhong et al. (2024); Tran et al. (2016); Rosen et al. (2016)
Nutritional Epigenetics	Impact of dietary factors on epigenetic marks (e.g., polyphenols, omega-3 fatty acids)	Diet-based interventions; Personalized nutrition plans based on epigenetic profiles	Prevention of obesity through sustained metabolic health and reduced adiposity	Siroma et al. (2022), Zhou et al. (2024); Abeltino et al. (2024)

## Chemical reprogramming and small-molecule compounds

Recent research reveals that the combinations of small-molecule compounds can reprogram human somatic cells into chemically induced pluripotent stem cells (CiPSCs). Unlike plasmid and viral transfections that require exogenous regulatory mediators, potentially integrating external factors and posing safety risks, the chemically induced methods are safer and easier to operate. CiPSC processes are more controllable and standardizable, advancing regenerative medicine. Chemical reprogramming considers both signaling pathway regulation and epigenetic effects in selecting compounds (Mitchell et al. 2024).

The use of small-molecule compounds in reprogramming offers several advantages over traditional methods. These compounds can target specific epigenetic modifiers, such as histone deacetylases (HDACs) or DNA methyltransferases, to facilitate the remodeling of the epigenome during reprogramming. Additionally, small molecules can modulate key signaling pathways involved in pluripotency and cell fate determination, such as the Wnt, TGF-β, etc. (Pappas et al. 2020). Some examples of small molecules used in chemical reprogramming include Scriptaid, an HDAC inhibitor that improves chromatin reprogramming after nuclear transfer (Macedo et al. 2022); 5-Azacytidine, a DNA methyltransferase inhibitor that enhances DNA demethylation and activation of pluripotency genes (Albany et al. 2017); CHIR99021, a specific inhibitor of GSK3β, induces Tcf7l1 protein degradation, which facilitates the maintenance of an undifferentiated state in mouse embryonic stem cells (Yu et al. 2024). Therefore, the combination of these and other small molecules can synergistically promote reprogramming by targeting multiple epigenetic and signaling pathways simultaneously.

Noticeably, chemical reprogramming, while offering a great advantages over traditional reprogramming methods, is not devoid of challenges and limitations, whose key points are outlined in Table 2.

**Table 2:** The succinct summary of the challenges and limitations of chemical reprogramming

Challenge/Limitation	Explanation	References
Complexity of Epigenetic Regulation	Small molecules may not fully replicate the complex interactions of native cellular signals, leading to incomplete reprogramming	Polak et al. (2016)
Off-Target Effects	Specificity of small molecules are compromised, interacting with unintended targets and causing unwanted phenotypic changes	Xiao et al. (2021) Tang et al. (2018)
Scaling Up	While controllable and standardizable, scaling chemical reprogramming for large-scale production of iPSCs or CiPSCs is challenging and requires optimization	Farzaneh et al. (2017)
Safety Concerns	Use of small molecules at high concentrations or over extended periods may lead to genotoxicity or uncontrolled cellular proliferation	Hajra et al. (2018)
Efficiency and Consistency	Chemical methods are not as efficient as viral or non-integrating episomal methods, with variable reprogramming outcomes	Chen et al. (2024)
Cost and Availability	The cost of small molecules and the need for multiple compounds are high, their availability and stability under different conditions limit the use	Wang et al. (2023); Liuyang et al. (2023)
Understanding of Molecular Mechanisms	The mechanisms of small molecules inducing reprogramming are not fully understood, which is crucial for optimizing protocols and predicting outcomes	Rehman et al. (2024)

## Advanced technologies for investigating epigenetic modifications

Current methods investigating epigenetic modifications in cell programming/reprogramming include high-throughput sequencing technologies, reduced representation bisulfite sequencing (scRRBS), single-cell DNA ChIP-seq, and single-cell RNA-seq (scRNA-seq). These technologies elucidated transcriptional and epigenetic (chromatin) level regulatory processes in cell differentiation (Schmolka et al. 2015; Stuart and Satija 2019).

Single-cell technologies have revolutionized our understanding of cellular heterogeneity and the dynamics of epigenetic regulation during development and reprogramming. scRNA-seq allows for the profiling of gene expression at the individual cell level, providing insights into the transcriptional changes that occur during cellular transitions (Yue et al. 2024). Similarly, single-cell epigenomic techniques, such as single-cell DNA methylation and single-cell ATAC-seq, enable the mapping of DNA methylation and chromatin accessibility patterns in individual cells, respectively (Danese et al. 2021). These advanced techniques have revealed the complex and dynamic nature of epigenetic regulation during cellular reprogramming, for example, studies using single-cell approaches have identified distinct epigenetic states and trajectories during iPSC generation, highlighting the heterogeneity and stochasticity of the reprogramming process (Wang et al. 2022).

Other methods for investigating epigenetic regulation in cell programming/reprogramming include the following approaches, CRISPR-Cas9 and other genome editing tools are used to modify specific epigenetic regulators and study their effects on cellular plasticity and the targeted mutagenesis (Yuan et al. 2020); Electrophoretic mobility shift assay (EMSA) is analyzed for protein–DNA interactions to identify transcription factors involved in epigenetic regulation (Kalra et al. 2022); Candidate gene ChIP is applied to investigate histone modifications and transcription factor binding at specific genomic loci (Cavalli et al. 2019); real-time fluorescent tagging of chromatin structures is showed in visualizing dynamic changes in chromatin organization during reprogramming (Sardo et al. 2017); Somatic cell nuclear transfer (SCNT) primary study the epigenetic reprogramming in the aspect of cloning and nuclear reprogramming (Li et al. 2022); Cell fusion can explore the epigenetic changes that occur when somatic cells are fused with pluripotent stem cells (Cantone et al. 2016); transcription factor and microRNA-induced pluripotency may analyze the epigenetic changes induced by specific reprogramming factors (Krishnakumar and Blelloch 2013). Thus, these diverse approaches provide complementary insights into the complex epigenetic mechanisms underlying cellular plasticity and reprogramming.

Noticeably, the challenges and limitations associated with these approaches are also concerned, i.e., scRNA-seq generates vast amounts of data, requiring sophisticated bioinformatics tools for analysis. The complexity of data processing is difficult for researchers without specialized computational expertise (Haque et al. 2017). Its limitation is sensitive to technical variability, such as differences in library preparation and sequencing depth, which can introduce biases into the data (Luecken and Theis 2019); ChIP requires a significant amount of high-quality chromatin and is technically demanding, with potential for low efficiency in immunoprecipitation, especially for histone modifications that are present at low abundance (Hu et al. 2022). Its limitation may not be suitable for all cell types or tissues, particularly those that are difficult to cross-link or shear, and it may not capture the dynamic nature of chromatin interactions (Kelley et al. 2017); SCNT is technically complex and has low success rates. It also raises ethical concerns, particularly when applied to human cells. The method is limited by the availability of oocytes and the potential for reprogramming errors, which can result in abnormal gene expression patterns (Shufaro and Reubinoff 2017); cell fusion is challenging to control the risk of generating heterokaryons that do not fully reprogram. Its limitation may not provide insights into the precise epigenetic changes that occur during reprogramming, as it involves the merging of two distinct cell types (Chen et al. 2006); the reprogramming efficiency of transcription factor and microRNA-induced pluripotency is variable, and the overexpression of transcription factors or microRNAs lead to uncontrolled cell proliferation or tumorigenesis. The limited use of viral vectors for introducing reprogramming factors result in insertional mutagenesis, and there are concerns about the immunogenicity of viral proteins (Masip et al. 2010); identifying the correct combination of small chemical molecules that can effectively induce reprogramming is challenging and often requires extensive screening. Chemical reprogramming may not be as efficient as other methods, and the long-term effects of small molecules on cellular epigenetics and genomic integrity are not fully understood (Wang et al. 2023; Liuyang et al. 2023); CRISPR-Cas9 can introduce off-target effects, and the precision of genome editing is influenced by factors such as guide RNA design and delivery methods. The technology requires highly specific conditions for optimal efficiency, and the potential for unintended genomic alterations poses risks for therapeutic applications (Tang et al. 2018).

## Future directions

The field of epigenetics is rapidly evolving, yet it faces several challenges that must be overcome to fully harness its potential in clinical applications. Firstly, current techniques such as scRNA-seq, ChIP, and bisulfite sequencing, while powerful, can be limited by their scalability, cost, and the depth of single-cell analysis. There is a need for more sensitive and high-throughput methods that provide comprehensive epigenomic profiles at the single-cell level; secondly, the integration of multi-omics data (epigenomics, transcriptomics, proteomics) presents a significant challenge due to the complexity and heterogeneity of biological systems. Developing algorithms and computational frameworks that can effectively integrate these diverse data types is crucial for a comprehensive understanding of cellular states; thirdly, the development of more precise and targeted epigenetic editing tools is necessary to manipulate specific epigenetic marks in a cell-type-specific manner. This will help to avoid off-target effects and improve the safety and efficacy of epigenetic therapies; fourthly, the mechanisms of transgenerational epigenetic inheritance are not fully understood and require further investigation. Understanding these mechanisms could have profound implications for human health and evolution; fifthly, translating epigenetic research into clinical practice presents regulatory, ethical, and logistical challenges. It is vital to establish standardized protocols and guidelines for the clinical application of epigenetic therapies; Sixthly, the current state of drug delivery systems, including nanoparticles and liposomes, must be improved to ensure bioavailability, specificity, and to minimize potential side effects; Finally, the influence of environmental and lifestyle factors on the epigenome is an emerging area of research, on the other hand, the large-scale screening has been instrumental in identifying genes and pathways susceptible to epigenetic control, particularly in the context of adipogenesis and metabolic homeostasis. Understanding these interactions could lead to novel preventive strategies and interventions.

## Conclusion

In summary, understanding the mechanisms behind cellular programming and reprogramming provides insights into novel therapeutic strategies for obesity. By manipulating these pathways, it may be possible to develop therapies that not only prevent obesity but also reverse its effects by altering the fundamental cellular processes involved in fat storage and metabolism, including a conversion from harmful fat-storing cells to beneficial fat-burning cells.

## Data Availability

No datasets were generated or analysed during the current study.

## Abbreviations

ABCG1: ATP-binding cassette subfamily G member 1
BAF60b: Brahma-related gene 1
BAT: Brown adipose tissue
C/EBPb: CCAAT-enhancer-binding protein beta
ChIP: Chromatin immunoprecipitation
CiPSCs: Chemically induced pluripotent stem cells
C-myc: Myc proto-oncogene protein
DNA: Deoxyribonucleic acid
DNMTs: DNA methyltransferases
DOHaD: Developmental origins of health and disease
Dppa2: Pluripotency-associated 2
Dppa4: Pluripotency-associated 4
ES cells: Embryonic stem cells
FTO: Fat mass and obesity-associated gene
H3K4me3: Histone H3 lysine 4 trimethylation
HDACs: Histone deacetylases
HDAC9: Histone deacetylase 9
iPSCs: Induced pluripotent stem cells
Klf4: Kruppel-like factor 4
LEP: Leptin
MC4R: Melanocortin 4 receptor
miR-27: MicroRNA-27
miR-33: MicroRNA-33
miRNAs: MicroRNAs
MHO: Metabolically healthy overweight/obesity
MUHO: Metabolically unhealthy overweight/obesity
ncRNAs: Non-coding RNAs
NEFA: Nonesterified fatty acids (free fatty acids)
Oct4: Octamer-binding protein 4
piRNAs: PIWI-interacting RNAs
POMC: Pro-opiomelanocortin
PPARG: Peroxisome proliferator-activated receptor gamma
Prdm9: Protein arginine methyltransferase 9
SCNT: Somatic cell nuclear transfer
scRNA-seq: Single-cell RNA sequencing
siRNAs: Small interfering RNAs
Sox2: SRY-Box transcription factor 2
SWI/SNF: Switch/sucrose-nonfermentable chromatin-remodeling complex
TET: Ten-eleven translocation methylcytosine dioxygenases
WAT: White adipose tissue
ZGA: Zygotic genome activation
